# Characterization of *Fusarium venenatum* Mycoprotein-Based Harbin Red Sausages

**DOI:** 10.3390/foods14040556

**Published:** 2025-02-07

**Authors:** Xue-Li Li, Xian-Ni Qi, Jia-Chen Deng, Ping Jiang, Shu-Yuan Wang, Xing-Li Xue, Qin-Hong Wang, Xiaoqing Ren

**Affiliations:** 1 College of Food Science and Bioengineering, Tianjin Agricultural University, Tianjin 300392, China; lixueli@tib.cas.cn (X.-L.L.); nonsense_123@sina.com (J.-C.D.); 18428364175@163.com (P.J.); wang2795713988@163.com (S.-Y.W.); 15249535767@163.com (X.-L.X.); 2 Key Laboratory of Engineering Biology for Low-Carbon Manufacturing, Tianjin Institute of Industrial Biotechnology, Chinese Academy of Sciences, Tianjin 300308, China; qi_xn@tib.cas.cn; 3 National Innovation Centre for Synthetic Biology, Tianjin 300308, China

**Keywords:** *F. venenatum* mycoprotein, red sausage, sensory evaluation, gas hromatography–ion mobility spectrometry (GC-IMS)

## Abstract

*Fusarium venenatum* mycoprotein is an alternative, nutritious protein source with a meat-like texture. Here, *F. venenatum* mycoprotein-based Harbin red sausage was developed and characterized. The study focused on the effect of mycoprotein on the quality of red sausages, which were evaluated in five groups of red sausages based on nutrient content, differential scanning calorimetry (DSC), and gas chromatography–ion mobility spectrometry (GC-IMS). The results showed that increasing the component of mycoprotein in red sausage increased the protein and volatile organic compound content but decreased the water and ash content. There was no significant difference (*p* > 0.05) between red sausage with 25% added mycoprotein and traditional red sausage in terms of redness and thawed water component, but the protein component was higher, the flavor substances were slightly richer, and the consumer preference was higher. These results suggest that moderate amounts of mycoprotein can improve nutritional value and maintain sensory quality, but that higher levels of substitution can adversely affect preference. This study highlights the potential of mycoprotein as an artificial meat that can strike a balance between improved nutritional value and sensory acceptability.

## 1. Introduction

Harbin red sausage, originally named RIDOS, from the Soviet Union, is a common eastern European family dish. Red sausage is fat but not greasy, nutrient-rich, and so on; the five most important steps in the processing technology are filling, salting, enema, cooking, and smoking; smoking the sausage will give it its red skin and wood-smoked incense quality characteristics [1]. Compared with other enema products, Harbin red sausage tastes more mellow and delicious and is chewy, flexible, and non-greasy. *F. venenatum* mycoprotein, as a highly efficient and nutritious source, has a far-reaching impact on reducing the environmental footprint of meat consumption, and the introduction of mycoprotein into traditional food products such as Harbin red sausages helps to enrich their flavor, improve their nutritional value, and achieve environmental recyclability, as well as integrating new elements for them in modern food culture. Through this combination, Harbin red sausage can not only maintain its cultural characteristics, reflecting the organic combination of food technology and cultural innovation, but also give new life to traditional foods, enhance the health value and environmental sustainability of the product, and, at the same time, adapt to the increasingly changing consumer demand for a healthier and more environmentally friendly future.

As the world population grows and living standards gradually improve, the pressure on meat supply is increasing. The world population is expected to increase to 9 billion by 2050, and the demand for meat will increase by more than 50%. *F. venenatum* mycoprotein has been developed to address the greenhouse gas emission, animal welfare, and human health problems associated with meat production. Mycoprotein is a kind of substitute protein produced by using renewable biomass as a substrate and cultivating microorganisms in a fermenter. Compared with traditional protein sources, the mycoprotein production process can obtain more protein with fewer resources and has many advantages such as high production efficiency and low carbon dioxide emission [2,3].

At present, the main single-cell protein is the mycoprotein obtained from the fermentation of *F. venenatum*, which has a meat-like structure and has been used to produce meat substitutes [4,5,6]. *F. venenatum* can make full use of industrial waste water and agricultural and sideline products as the reaction substrate to provide carbon and nitrogen sources so that the cost of production can be effectively controlled; the protein content of mycoprotein is more than 40%, the amino acids are balanced, and it is rich in dietary fiber, vitamins and minerals [7,8]. According to Zhang Xuewen’s [9] analysis, the protein content of mycoprotein accounted for 56.65% of its dry weight, which was much higher than that of soybean (38.01%) and hericium erinaceus (15.04%), and the dietary fiber content of mycoprotein was 32.95%, which is 1.4 times more than soy and 5.4 times more than hericium erinaceus, and the unsaturated fat in mycoprotein makes it a healthy choice [10]. Studies have shown that mycoprotein added to meat products can effectively reduce the cooking loss rate and increase its water retention and juiciness [9].

## 2. Materials and Methods

### 2.1. Materials

*F. venenatum* mycoprotein was provided by the Tianjin Institute of Industrial Biotechnology (Tianjin, China). The mycoprotein was processed by first pulverizing it into dry powder with a universal pulveriser (FW-80, Beijing Yong Guangming Medical Instrument Factory, Beijing, China) and passing it through an 80-mesh sieve; then, in a food processor (3205FP3010, De’Longhi Braun Hpuserhold GmbH, Debrecen, Hungary), 3 parts of mycoprotein and 7 parts of water were added to make mixed mincemeat; these steps are more suitable for red sausage processing. Lean pork, salt, sugar, egg, corn starch, and white pepper were purchased from Yonghui Supermarket; pig intestines were purchased from the Beijing Dongdajie self-owned flagship shop (Longda Meat Food Co., Ltd., Beijing, China); compound phosphate (food-grade), sodium ascorbate (food-grade), and TG enzyme (food-grade) were purchased from the Taobao Jiahe Xueri Flagship Store (Shanghai Jizhi Biochemical Technology Co., Ltd., Shanghai, China); and sodium nitrite (food-grade) was purchased from the Taobao Guanyuejia Flagship Store (Beijing Solaibao Technology Co., Ltd., Beijing, China).

### 2.2. Sausage Preparation

Three batches of sausages were made independently (replicates), and each batch produced a total of five sets of sausages by mixing (0%, 25%, 50%, 75%, 100%) mycoprotein with (100%, 75%, 50%, 25%, 0%) lean pork meat, which were named as pure meat sausage (Mp1), 25% *F. venenatum* mycoprotein-based Harbin red sausage (Mp2), 50% *F. venenatum* mycoprotein-based Harbin red sausage (Mp3), 75% *F. venenatum* mycoprotein-based Harbin red sausage (Mp4), and 100% *F. venenatum* mycoprotein-based Harbin red sausage (Mp5). The basic recipe for red sausage is mapped out by preliminary experiments and is as follows: 2.5 kg lean pork, 50 g salt, 7.5 g compound phosphate, 1.5 g sodium ascorbate, 0.25 g sodium nitrite, 10 g sugar, 10 g dextrose, 100 mL water, 200 g maize starch, 50 g minced garlic, 6 g white pepper, 200 g egg, 10 g white wine (50°), and 400 g iced water.

Lean pork was minced with a meat grinder (BJRJ-82, Zhejiang Jiaxing Aibo Industry Co., Ltd., Jiaxing, China) and marinated with marinade. Meat was processed in a food processor, and mycoprotein was mixed with pork, egg, white pepper, garlic, ice water, and TG enzyme, and stirred to obtain mycoprotein red sausage meat filling. *F. venenatum* mycoprotein-based Harbin red sausage was put into an enema machine (XZ-5L, Guangzhou Xuzong Food Machinery Co., Ltd., Guangzhou, China) for enema, splitting, and ventilating, and then put into a multifunctional smoking oven (BYXX-50, Zhejiang Jiaxing Aibei Industry Co., Ltd., Jiaxing, China) for baking (65 °C, 1 h), steaming (85 °C, 1 h), and smoking (60 °C, 2 h), and then finally five groups of red sausages were obtained.

### 2.3. Nutrient Content Analysis

Chopped samples (3 g each) were dried in an oven at 105 °C for 2–4 h, then cooled to room temperature in a desiccator and weighed; drying, cooling and weighing were repeated, and moisture content was calculated from the difference in mass before and after oven drying [11]. A 4 g sample was weighed and burnt in a muffle furnace at 525 °C until constant weight and the ash content was obtained from the weight of the measured ash [12]. According to the Kjeldahl method to determine the protein content, 0.5 g of the sample was weighed in the Kjeldahl apparatus supporting the test tube, accurate to 0.001 g; we added concentrated HSO4 to facilitate digestive furnace digestion. After the digestion process was over, we cooled and removed the semi-automatic Kjeldahl apparatus, removing the receiving bottle as soon as possible, and turned to the titration stage to reach the end point of sulfuric acid titration of the standard solution; we then performed a calculation of the results [13]. The samples were weighed in folded filter paper, placed in a Soxhlet extractor with a capacity of 250 mL, and heated with petroleum ether as the solvent on a water bath for 6 h. After extraction, the filter paper was placed in a vacuum desiccator until a constant weight was reached, and the fat content was calculated by the difference in the mass of the filter paper [14].

### 2.4. Color Measurement

Color measurements were performed by a colorimeter (HP-2132 Portable Colorimeter, Hanpu Color Technology Co., Shenzhen, China) using a D 65 light source and a 10°observer with an 8 mm diameter measuring area and a 50 mm diameter illumination area. A white standard plate (L* = 149 95.26, a* = −0.89, b* = 1.18) was used for calibration prior to measurements. Results were obtained from three different parts of the sausages as L* value (lightness), a* value (redness) and b* value (yellowness), and the average value was recorded. The instrument operating instructions guided the experimental operations.

### 2.5. DSC Analysis

According to the method of Rozita Vaskoska [15] and some modifications, we weighed the sample to about 10 mg and placed the cap on aluminum foil. The parameters of DSC (DSC 200 F3, NETZSCH, Selb/Bavaria, Germany) were as follows: purge gas 2 MFC, nitrogen, flow rate of 60 mL/min; protective gas MFC, nitrogen, flow rate of 60 mL/min; temperature: −20 °C~100 °C, heating at 5 °C/min; emergency protection temperature: 110 °C. At the end of the experiment, the temperature was decreased by 40 °C/min.

The content of unfrozen water is equal to the difference between the water content minus the frozen water content, and unfrozen water includes bound water and unbound water. Sausages contain almost no bound water; therefore, the content of non-frozen water is mainly unbound water [16]. The melting enthalpy of frozen water can be calculated by integrating the peak value of the integral melting curve. The melting enthalpy is the area of the curve below the peak.

The frozen water content formula is as follows:

(1) $$W_{f}=\frac{{∆H}_{f}}{∆H_{m}}\times 100\%$$

In the formula, *W_f_* represents the content of frozen water; Δ*H_f_* represents the melting enthalpy of frozen water; Δ*H_m_* represents the standard pure water melting enthalpy of 334 J/kg.

The formula for unfrozen water is as follows:

(2) $$W_{n}=W_{c}-W_{f}$$

In the formula, *W_n_* stands for Unfrozen Water Content; *W_c_* stands for water content; *W_f_* stands for frozen water content.

### 2.6. Sensory Analysis

The sensory evaluation of 5 groups of *F. venenatum* mycoprotein-based Harbin red sausage was carried out according to the ranking test. All participants in the sensory evaluation experiment signed informed consent forms. The participants were all first- or second-year postgraduate students of Tianjin Agricultural College, majoring in food, and aged between 23 and 25 years old. The samples were presented to 10 reviewers in an equilibrium random order. The reviewers were asked to rank the samples according to their preferences [17,18,19]; we calculated the sum of the sequences and then used the Friedman method to analyze the data. Mp5, Mp4, Mp3, Mp2, and Mp1 were named A, B, C, D, and E, respectively. The sorting test question-and-answer table is shown in Table 1.

The statistic f is calculated using the Friedman test, and the formula is as follows:

(3) $$F=\frac{12}{JP\left(P+1\right)}\left(R_{1}^{2}+R_{2}^{2}+\ldots +R_{P}^{2}\right)-3J\left(P+1\right)$$

*J* stands for the number of reviewers; *P* stands for the number of samples; *R*_1_, *R*_2_, …, *R_P_* stand for the rank sum of each sample.

If a reviewer cannot tell the difference between samples, we revise the statistic *F* value, expressed as *F*’.

(4) $$F^{\prime }=\frac{F}{1-\frac{E}{\left[JP\left(P^{2}-1\right)\right]}}$$

(5) $$E=\left(n_{1}^{3}-n_{1}\right)+\left(n_{2}^{3}-n_{2}\right)+\ldots +\left(n_{k}^{3}-n_{k}\right)$$

*F*’ indicates the value of correction; *J* represents the number of reviewers; *P* indicates the number of samples; *n*_1_, *n*_2_, …, *n_k_* indicate the number of samples with the same rank.

The critical value was calculated according to the rank sum of each sample, and line analysis was carried out according to the comparison between the rank sum difference and the critical value of each sample.

Calculation of the critical value *r*(*I*,*α*):

(6) $$r\left(I,\alpha \right)=q\left(I,\alpha \right)\sqrt{\frac{JP\left(P+1\right)}{12}}$$

*J* stands for the number of reviewers; *P* stands for the number of samples.

### 2.7. Electronic Nose Analysis

According to the proposal of Chen Yuanyan [20,21], minor modifications were made in which 5 g samples were taken from 5 groups of *F. venenatum* mycoprotein-based Harbin red sausage and crushed in a 20 mL headspace bottle. The samples were bathed in a 50 °C water bath for 20 min, followed by manual injection into the Heracles II ultrafast electronic nose (Heracles II Ultra-Fast Electronic Nose, Alpha M.O.S, Toulouse, France); the instrument analysis parameters are shown in Table 2.

### 2.8. GC-IMS Analysis

Rapid, nondestructive, high-throughput testing and screening of volatile ingredients plays an important role in food flavor analysis. GC-IMS is a powerful technique for the separation and sensitive detection of volatile organic compounds [22]. Flavor profiling was carried out using a FlavourSpec GC-IMS instrument from G.A.S., Inc. (Dortmund, Germany). The instrument was equipped with an FS-SE-54-CB-1 column (15 m × 0.53 mm × 1.0 µm) with a controlled column temperature of 60 degrees Celsius, using nitrogen as the carrier gas [23]. Three *F. venenatum* mycoprotein-based Harbin red sausages of similar size were selected for each treatment group for parallel experiments. Briefly, 0.5 g of sample was sealed in a 20 mL headspace vial and 200 µL of headspace vapor was injected into the GC and incubated for 20 min at 50 °C [24]. The IMS temperature was 45 °C and the analysis time was 30 min. Three analyses were carried out for each sample and retention indices (RIs) were determined. Identification of volatile compounds was completed by comparing the RIs with those of known standards in the GAS GC-IMS library. Flavor compounds were qualitatively analyzed using Laboratory Analytical Viewer (LAV) software (v2.2.1, G.A.S., Dortmund, Germany) in conjunction with the GC-IMS database in the Matches folder in the GC-IMS Library Search software (v1.0.1, G.A.S., Dortmund, Germany).

### 2.9. Statistical Analysis

Three parallel sets of each of the five treatment groups were tested in each experiment (nutrient analysis, color, DSC, sensory analysis, electronic nose, GC-IMS). Data were expressed as mean ± standard deviation and were analyzed using Microsoft Excel 2022 for table plotting and Adobe Photoshop CC 2019 for image modification; one-way analysis of variance (ANOVA) was performed using IBM SPSS Statistics 25 software at a significance level of *p* < 0.05, and Tukey’s method was used for multiple-group analysis of variance.

For electronic nose data analysis, PCA analysis was performed using Origin 2021 software. For GC-IMS data analysis, the LAV analysis software (v2.2.1, G.A.S., Dortmund, Germany) and plug-ins (Galery, Reporter, etc.) accompanying the flavor analyzer were used to perform the difference analysis of the plots, and the GC-MS Library Search database built into the application software was used for the qualitative analysis of the substances.

## 3. Results

### 3.1. Effect of F. venenatum Mycoprotein on Nutritional Components of Red Sausage

**Figure 1:**
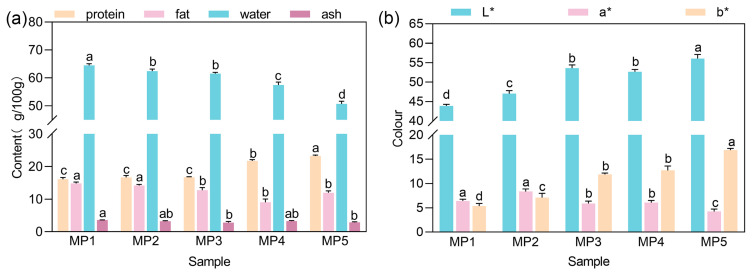
Effect of different *F. venenatum* mycoprotein additions on (**a**) nutritional components and (**b**) color of red sausage. Note: different letters indicate significant difference (*p* < 0.05).

The basic nutritional components of the five groups of red sausages produced with mycoprotein in place of the lean pork meat are shown in Figure 1a. According to the standard of T/HHCX 0001-2018 Harbin red sausage, the modified Harbin red sausage requires moisture (g/100 g) ≤ 65 and protein (g/100 g) ≥ 16. The five groups of red sausages that were produced in this trial all complied with the requirements of the standard (Table S1). A similar result has been reported by Xiaoyu Yin et al. [25]. With the increase in mycoprotein substitution, the protein content gradually increases, which is conducive to improving human metabolism and enhancing immunity, while fat, ash and water contents gradually decrease; reducing fat content can avoid health problems caused by obesity, so the red sausage is in line with the pursuit of health and nutritional needs via consumption of the people.

### 3.2. Effect of F. venenatum Mycoprotein on Color Difference in Red Sausage

Color is an important index that is used to evaluate the freshness of meat products [26]. As can be seen from Figure 1b, with the increase in the replacement amount of mycoprotein, the color difference in red sausage showed a trend of decrease in the value of red and an increase in the value of brightness. However, there is no significant difference (*p* > 0.05) between Mp2 and Mp1, because the color of mycoprotein is grayish yellow and the color of pork is red; in the case of a small amount of substitution of 25%, the a* value of red sausage is basically stable, and as the amount of substitution is increased, the original color of pork is affected by mycoprotein, so the a* value becomes smaller. Combined with the changes in L*, a*, and b* values (Table S2), it can be concluded that the color of the sausage was related to the amount of mycoprotein.

### 3.3. DSC Analysis of F. venenatum Mycoprotein Red Sausage

DSC is a common experimental instrument used to study the thermal properties of substances during heating or cooling [27,28]. DSC can measure the energy difference in absorption or exothermic reactions of a sample at different temperatures and obtain the thermal analysis data of the material, including the melting point, crystallization behavior, glass transition temperature, etc. [29,30].

**Figure 2:**
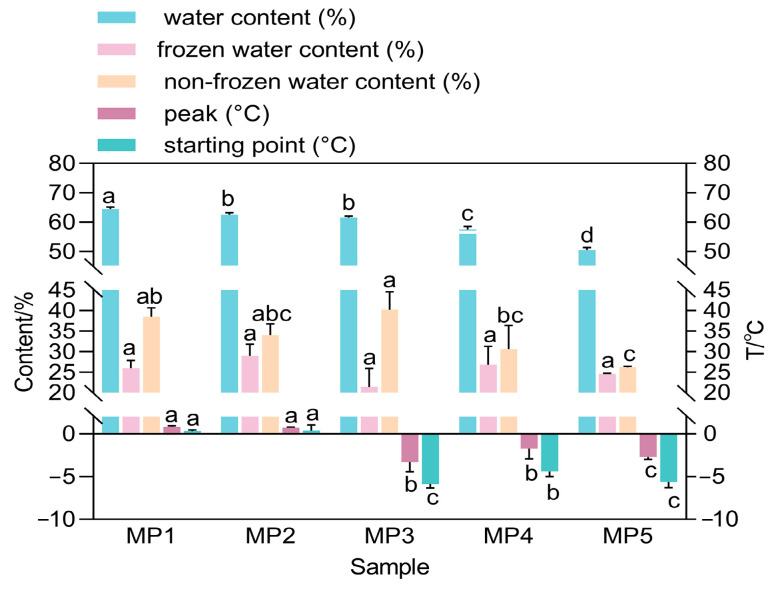
Effect of addition of different amounts of *F. venenatum* mycoprotein on DSC of red sausage. Note: different letters indicate significant difference (*p* < 0.05).

Low, medium, and high moisture contents of a food product can affect the way the food is processed or stored. By investigating the effect of maltodextrin addition on the freezing point and glass transition temperature required for the construction of state diagrams of a model system for fruit juices by differential scanning calorimetry, a mathematical model based on the composition of the solutes was developed to assess the effect of chemical composition on the thermal transition and to predict the state diagrams of the juices at different maltodextrin mass fractions [31]. The aim of this study was to investigate the effect of ultrasound-assisted immersion freezing, immersion freezing, and air freezing on the quality, moisture distribution, and microstructural properties of minced pork during storage. DSC analysis showed that changes in moisture distribution in the samples could be measured, which could help to improve product quality satisfaction [32]. The water content, frozen water content, and non-frozen water content of the five groups are shown in Figure 2. In terms of the water content (Table S3), there is a decreasing trend as the amount of mycoprotein substitution increases, which will increase the firmness and chewiness of the red sausage, and a low water content means that the concentration of other ingredients (e.g., fats, proteins, etc.) is relatively high, which will help to increase the concentration of flavor in the sausage, making it more flavorful and potentially prolonging the shelf-life of the red sausage. Water binding and release are closely related to the texture of sausages, and sausages with good water-holding capacity usually have better tenderness and texture. The water-holding capacity of red sausage was assessed by DSC results. High thawing water content indicated that the samples had good water-holding capacity, and the thawing water content decreased with the increase in mycoprotein substitution, and there was no significant difference (*p* > 0.05) between Mp2 and Mp1, and there was no significant difference in tenderness and texture if the ability to hold water was basically the same during freezing, which also verified the results of the sensory ratings.

### 3.4. Effect of F. venenatum Mycoprotein on Sensory Evaluation of Red Sausage

The order of the reviewers’ preference for the five groups of sausages is shown in Table 3, and the rank and rank sum of the samples are shown in Table 4.

The ranking test in sensory evaluation allows for the sensory evaluation of differences between products by the sensory evaluator, depending on the nature or preference of the product [33]. Compared with other sensory evaluation methods, the sorting test method is simple and easy, and the requirement for equipment is not high [34].

Taking the data from Table 3 and Table 4 into the formula yields a statistic F of 38.2.

According to the calculations,

E = (23 − 2) + (23 − 2) + (23 − 2) = 18;

F’ = 38.78.

The results of Friedman’s rank sum test showed that the critical values of p, j, and α (5, 10.01) were 12.38, which were less than the modified statistic value of 38.78, and the difference was significant (*p* < 0.01).

**Figure 3:**
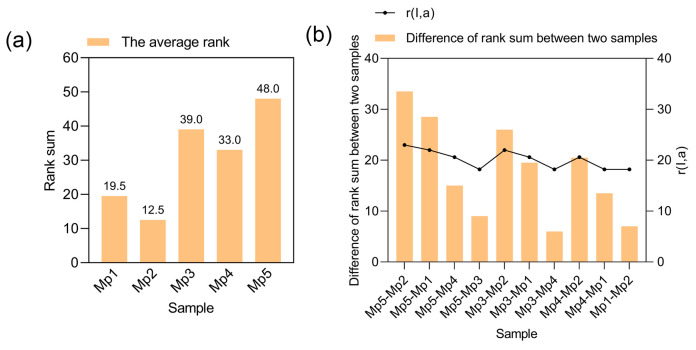
Rank sum (**a**) and difference in rank sums (**b**) for red sausage samples based on ranking tests.

The horizontal coordinates are the samples and the vertical coordinates are the rank sums in Figure 3a; after concluding that the samples are highly significantly different using Friedman’s test, the smaller the rank sums, the higher the preference of the samples. The difference between the rank sums of the samples is calculated and compared to the corresponding critical value. If the difference between the rank sums of the two samples is less than the corresponding r value, it means that there is no significant difference between these two samples and all the samples whose rank sums lie between these two samples. If the difference between the rank sums of the two samples compared to each other is greater than the corresponding r value, then there is a significant difference between these two samples and all samples whose rank sums lie between these two samples. In Figure 3b, the samples can be divided into three groups, the first group being Mp2 and Mp1, the second group being Mp4, and the third group being Mp3 and Mp5.

Overall preference evaluation is also used for various types of food products, such as using overall preference to evaluate shredded cheddar cheese, which can measure the factors influencing consumer acceptance and willingness to purchase, while optimizing the intrinsic sensory characteristics of shredded cheddar cheese [33]. By measuring consumer preferences for plant-based-milk coffee and milk coffee samples to see if there was a significant difference in overall preference between samples, it was found that consumer preference for plant-based-milk coffee was not only dependent on the plant variety, but also highlighted the importance of enhancing the desirable attributes and reducing the undesirable attributes of each ingredient type [34]. The overall preference evaluation was also applied to red sausage products, and a rank order test of preference for the five types of red sausage revealed that there was a significant difference in taster preference for the five samples, with Mp2 being the most popular, followed by Mp1 and then Mp4, Mp3, and Mp5; when comparing and grouping the samples, it was found that there was no significant difference in the preference of tasters for either Mp2 or Mp1 (level of significance of 0.01); therefore, replacing lean pork with 25% mycoprotein does not affect the taste of red sausage, but adding too much can affect people’s evaluation of red sausage.

### 3.5. The Results of Electronic Nose Analysis of F. venenatum Mycoprotein Red Sausage

**Figure 4:**
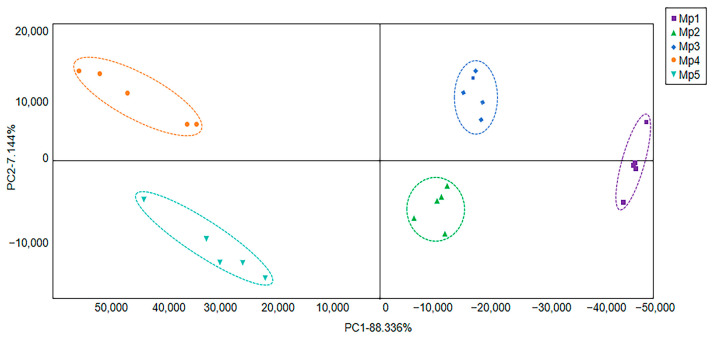
Principal component analysis via electronic nose of red sausage with different amounts of *F. venenatum* mycoprotein.

An e-nose is used as an alternative and rapid tool to characterize the complex odor patterns in various foods by mimicking the sense of smell and generating a sensor array response to a complete volatile pattern [35]. PCA is a statistical tool used to explain differentiation between samples and to extract information from the variables that mainly affect the sample spatial distribution [36]. Flavorings play a key role in determining the organoleptic properties of instant starch noodles, and the electronic nose can be used to classify different brands of instant starch noodle flavorings [37]. PCA has been used to show the initial relative position of the samples in two dimensions in order to observe changes in the volatiles of potato samples by means of an electronic nose, which was found to respond well to the odor of the species during the storage period [38]. PCA can be used to differentiate between VOCs identified from four species of silver carp, perch, bighead carp, and bream, and the results show that the odor profiles of raw and cooked fish are significantly different [39]. The results of PCA analysis of the volatile flavor compounds in the five groups of sausage samples are shown in Figure 4. Figure 4 reflects that the variance contribution rate of the first principal component was 88.336%; the variance contribution rate of the second principal component was 7.144%; and the total cumulative contribution rate of both was 95.48%. The results show that this map can reflect the integrity of the odor data of the samples. In PCA maps, the closer the relative distances between the samples, the more similar the overall odors of the samples, and the greater the difference if not [40]. As can be seen from the PCA profiles, there was no overlap between the five groups of samples, indicating that the Heracles II ultrafast gas-phase e-nose was able to distinguish the five groups of samples. The differences in odor among the five groups were mainly due to the different amounts of mycoprotein added.

### 3.6. GC-IMS Analysis of F. venenatum Mycoprotein Red Sausage

#### 3.6.1. Gas Chromatography–Ion Mobility Spectrometry (GC-IMS) Analysis

**Figure 5:**
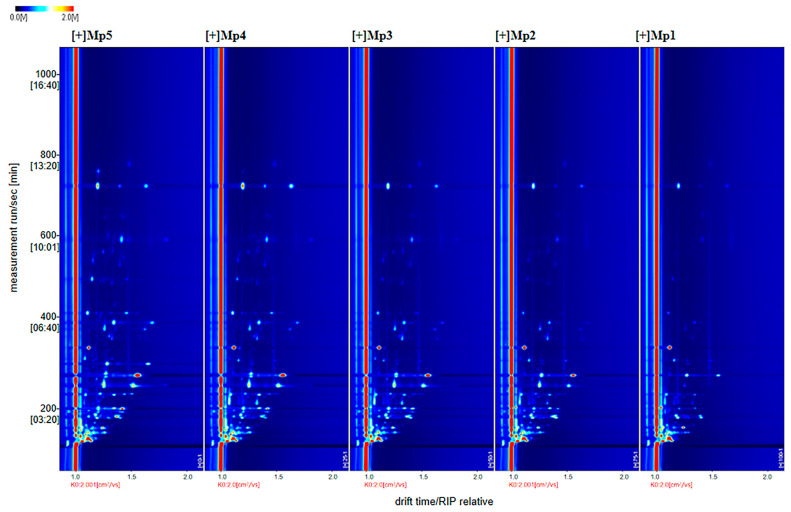
Two-dimensional spectra of red sausages with different *F. venenatum* mycoprotein amounts.

The two-dimensional GC-IMS map of *F. venenatum* mycoprotein-based Harbin red sausage is shown in Figure 5. The whole spectrum has a blue background, and the red vertical line at 1.0 is the Rip Peak (reactive ion peak, normalized) [41]. The vertical coordinates of the two-dimensional map represent the gas chromatographic retention time (s); the horizontal coordinates represent ion migration time (normalized); each point on either side of the RIP peak represents a volatile organic compound; and the color represents the concentration of the substance: white indicates lower concentrations, red indicates higher concentrations, and darker colors indicate higher concentrations [42]. From left to right, the volatile organic compounds were 100%, 75%, 50%, 25%, and 0%, respectively. From the graph, it can be seen that the different groups of samples have different types and contents of mycoprotein, and the higher the replacement amount of mycoprotein, the more volatile the sausage.

#### 3.6.2. Fingerprinting Analysis

**Figure 6:**
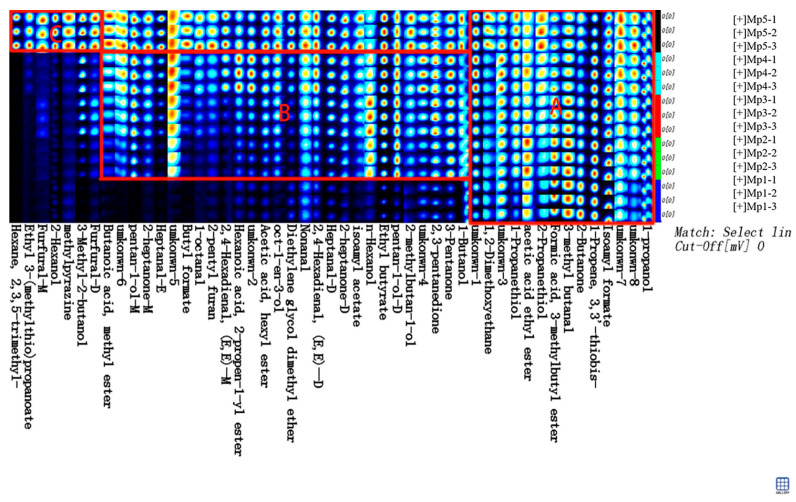
The fingerprint of red sausages with different amounts of *F. venenatum* mycoprotein. Note: Region A is for substances common to all five groups of intestines, region B is for substances not contained in Mp1, and region C is for substances unique to Mp5.

To more comprehensively analyze the flavor differences of different groups of sausage with mycoprotein, the fingerprint of five groups of sausage was obtained by using the Galerie plug-in in the LAV software of GC-IMS in Figure 6. With the increase in the replacement amount of mycoprotein, the content of flavor substances in the *F. venenatum* mycoprotein-based Harbin red sausage also increases. Region A is the volatile compound of the sausage with mycoprotein, such as n-propanol, isoamyl formate, and diallyl sulfide, while Region B is the flavor compounds added after the addition of mycoprotein; these are N-butanol, 3-pentanone, n-pentanol (monomer, dimer), etc., and they include allyl hexanoate, ethyl 3-methylthio propionate, furfural (monomer, dimer), etc.

A total of 49 peaks were detected in the samples, and 41 compounds were identified by the GC-IMS database in the Matches folder of the GC-IMS Library Search software, as shown in Table 5. Nine other volatile compounds were not identified due to incomplete flavor database information, which needs to be further identified in combination with other methods and literature reports.

The flavor substances were qualitatively analyzed using GC-IMS, including 11 alcohols, 9 esters, 9 aldehydes, 5 ketones, 4 sulfur-containing compounds, 1 pyrazine, 1 heterocyclic compound, and 1 alkane, as shown in Table 5. Aldehydes, which are important compounds in meat products, are formed by the thermal degradation of lipids [43,44]. Diallyl sulfide is the main flavor of garlic [45]; garlic flavor is the characteristic flavor of red sausage. The 2-methyl pyrazines of pyrazines have a toasty flavor and are the result of a smoky flavor [46]. The heterocyclic compound 2-pentylfuran is produced by the Maillard reaction and contributes to the greasy flavor of sausage [47]. Low concentrations (20 μmol/L) of furfural can reduce the production of reactive oxygen species, and high concentrations of furfural may induce cytotoxicity and organotoxicity [48]. Nevertheless, furfural compounds are widely distributed in foods to the extent permitted by national standards and can be used to flavor, preserve, and enhance the taste of foods [49]. According to the fingerprint, the peak color of furfural (monomer, dimer) in the red sausage with Mp5 was dark red. The peak color of furfural (monomer, dimer) became lighter gradually with the decrease in the replacement amount of mycoprotein, and the peak color of furfural (monomer, dimer) was black in the red sausage with the additional amount of mycoprotein; this indicates that furfural (monomeric, dimer) was not found in the red sausage added with mycoprotein. Because GC-IMS cannot be quantified, the content of specific flavor substances needs further study. It is inferred that the flavor of Mp2 is similar to that of Mp1 but slightly more abundant; this slightly rich flavor may be one of the reasons why sensual people prefer the Mp2 replacement to traditional red sausage.

After studying the effect of mycoprotein on red sausage by color, DSC, compositional analysis, etc., the results showed that the protein content and flavor substances of red sausage were effectively improved with the increase in mycoprotein substitution, and according to the results of organoleptic evaluation, 25% *F. venenatum* mycoprotein-based Harbin red sausage was preferred over the traditional red sausage. Overall, the favorable effect of mycoprotein on red sausage was verified by methods such as GC-IMS, which further demonstrated the feasibility of mycoprotein in the application of meat products such as red sausage, which may provide a theoretical basis for practical strategies of mycoprotein incorporation.

## 4. Conclusions

After studying the effect of *F. venenatum* mycoprotein on red sausage by color, DSC, compositional analysis, etc., the results showed that the protein content and flavor substances of red sausage were effectively improved with the increase in mycoprotein substitution, and according to the results of sensory evaluation, the 25% mycoprotein red sausage was preferred over the traditional red sausage. The application of mycoprotein in Harbin red sausage reflects the organic combination of food technology and cultural innovation, which gives new vitality to traditional food and enhances the health value and environmental sustainability of the product. Through this combination, Harbin red sausage can not only maintain its cultural characteristics, but also adapt to the ever-changing consumer demand and welcome a healthier and more environmentally friendly future. In conclusion, the qualitative analysis of the flavor substances of red sausage by GC-IMS showed that mycoproteins are beneficial to increase the flavor substances of red sausage, which further proves the feasibility of mycoproteins in the application of meat products such as red sausage and can provide a theoretical basis for microbial protein applications.

## Figures and Tables

**Table 1:** Question-and-answer sheet of sausage preference sorting test.

Name:		Date:		Products:	
Test instructions:	(1) samples A, B, C, D, E were sampled from left to right; (2) please judge the color, aroma, taste and shape of the sausage, rank the following products by how much you like them, from 1 very much to 5 very little.				
	1:	2:	3:	4:	5:

**Table 2:** Experimental conditions of Heracles II ultrafast gas-phase electronic nose.

Parameter	Optimization Conditions
Inlet temperature	200 °C
Injection duration	40 s
Initial trap temperature	40 °C
Trap shunting rate	10 mL/min
Final trap temperature	200 °C
The initial temperature of the column	50 °C
The programmed heating mode of column Temperature	0.5 °C/min~100 °C,1 °C/min~200 °C
Collection time	140 s
FID detector temperature	260 °C

**Table 3:** Rating results of reviewers.

Tasting Judges	The Order of Samples								
	1		2		3		4		5
1	D		E	=	B		A		C
2	E	=	D		C		B		A
3	D		E		C		B		A
4	D		E	=	B		A		C
5	D		E		C		B		A
6	D		E		A		C		B
7	E		D		B		C		A
8	D		E		C		B		A
9	D		B		E		C		A
10	E		D		B		C		A

Note: = is the same preference for both samples.

**Table 4:** Rank and rank sum of samples.

Tasting Judges	The Rank of the Sample					Rank Sum
	A	B	C	D	E	
1	4	2.5	5	1	2.5	15
2	5	4	3	1.5	1.5	15
3	5	4	3	1	2	15
4	4	2.5	5	1	2.5	15
5	5	4	3	1	2	15
6	5	5	4	1	2	15
7	5	3	4	2	1	15
8	5	3	4	1	2	15
9	5	2	4	1	3	15
10	5	3	4	2	1	15
Rank sum R for each sample	48	33	39	12.5	19.5	150

**Table 5:** GC-IMS analysis of volatile compounds in red sausages with different amounts of *F. venenatum* mycoprotein.

Number	Volatile Matter	Retention INDEX	Retention Time/s	Migration Time	Peak Strength				
					Mp1	Mp2	Mp3	Mp4	Mp5
Alcohols									
1 *	oct-1-en-3-ol	982.7	549.12	1.16	76.04 ± 7.64 ^e^	218.49 ± 8.36 ^d^	325.31 ± 2.41 ^c^	385.87 ± 14.90 ^b^	527.91 ± 22.45
2 *	Diethylene glycol dimethyl ether	960.3	503.37	1.14	271.70 ± 13.66 ^c^	196.00 ± 16.27 ^d^	260.58 ± 3.92 ^c^	515.34 ± 6.09 ^b^	1023.53 ± 18.09 ^a^
3 *	n-Hexanol	871.3	357.82	1.32	88.66 ± 1.68 ^e^	497.93 ± 21.88 ^b^	574.11 ± 12.50 ^a^	454.46 ± 3.61 ^c^	373.73 ± 14.80 ^d^
4 *	2-Hexanol	819.3	296.35	1.28	255.76 ± 8.63 ^c^	282.86 ± 12.81 ^c^	294.84 ± 8.21 ^c^	378.74 ± 2.78 ^b^	1787.11 ± 43.50 ^a^
5 *	pentan-1-ol-D	767.8	245.7	1.25	404.42 ± 14.65 ^e^	2549.14 ± 7.27 ^d^	2995.12 ± 18.09 ^c^	3120.45 ± 8.50 ^b^	3232.69 ± 32.84 ^a^
6 *	pentan-1-ol-M	763	241.45	1.52	87.55 ± 7.64 ^e^	1349.24 ± 22.77 ^d^	2055.40 ± 32.40 ^c^	2500.90 ± 13.78 ^b^	2803.21 ± 93.60 ^a^
7 *	2-methylbutan-1-ol	739.8	221.73	1.22	113.40 ± 4.91 ^e^	263.11 ± 7.87 ^d^	359.12 ± 2.13 ^c^	410.94 ± 6.83 ^b^	600.34 ± 7.26 ^a^
8 *	1,2-Dimethoxyethane	644.3	164.34	1.29	255.42 ± 3.79 ^c^	309.17 ± 13.26 ^b^	282.12 ± 3.21 ^b^	287.06 ± 4.47 ^b^	494.59 ± 30.25 ^a^
9 *	1-Butanol	651.5	167.34	1.37	678.84 ± 14.04 ^e^	1385.13 ± 48.36 ^b^	1285.09 ± 10.54 ^c^	1032.22 ± 30.26 ^d^	1543.17 ± 84.86 ^a^
10 *	1-propanol	543.6	127.36	1.11	3597.01 ± 19.01 ^c^	3844.56 ± 27.59 ^b^	3405.23 ± 21.62 ^d^	3967.10 ± 14.25 ^a^	3617.92 ± 8.95 ^c^
11 *	3-Methyl-2-butanol	692.5	186.46	1.41	72.22 ± 7.66 ^e^	364.63 ± 38.51 ^d^	1131.39 ± 43.76 ^c^	1377.97 ± 18.64 ^b^	2179.57 ± 53.70 ^a^
Esters									
12 *	Acetic acid, hexyl ester	1008.9	600.29	1.41	327.57 ± 23.17 ^e^	574.67 ± 39.38 ^d^	953.89 ± 9.09 ^c^	1227.49 ± 8.05 ^b^	1575.63 ± 32.23 ^a^
13 *	isoamyl acetate	874	361.36	1.3	47.25 ± 1.67 ^d^	150.76 ± 4.05 ^c^	237.18 ± 9.77 ^b^	255.99 ± 10.25 ^b^	343.89 ± 21.34 ^a^
14 *	Isoamyl formate	796.4	272.77	1.27	782.84 ± 12.19 ^d^	1108.35 ± 5.16 ^c^	1220.96 ± 16.37 ^b^	1248.93 ± 7.73 ^a^	1269.77 ± 16.81 ^a^
15 *	Ethyl butyrate	790.1	266.58	1.56	916.09 ± 20.60 ^e^	2968.30 ± 123.17 ^d^	4582.01 ± 32.62 ^c^	5603.71 ± 13.79 ^b^	6684.60 ± 150.21 ^a^
16 *	Formic acid, 3-methylbutyl ester	786.9	263.49	1.27	648.66 ± 24.47 ^d^	837.37 ± 18.33 ^a^	787.03 ± 11.14 ^b^	730.20 ± 10.01 ^c^	645.82 ± 17.13 ^d^
17 *	Butanoic acid, methyl ester	738.8	220.96	1.44	11.21 ± 1.05 ^e^	54.51 ± 11.21 ^d^	96.07 ± 0.56 ^c^	138.40 ± 5.82 ^b^	208.65 ± 12.29 ^a^
18 *	Butyl formate	740.2	222.12	1.5	9.55 ± 0.24 ^e^	30.09 ± 2.43 ^d^	61.04 ± 2.60 ^c^	92.93 ± 3.08 ^b^	232.06 ± 15.63 ^a^
19 *	acetic acid ethyl ester	596.3	145.52	1.09	219.77 ± 4.46 ^b^	226.88 ± 4.20 ^a^	199.67 ± 11.58 ^c^	217.47 ± 5.84 ^b^	202.24 ± 13.47 b^c^
20 *	Hexanoic acid, 2-propen-1-yl ester	1077.9	729.53	1.4	129.16 ± 24.66 ^e^	216.38 ± 12.84 ^d^	308.66 ± 24.66 ^c^	660.19 ± 18.95 ^a^	479.77 ± 660.19 ^b^
Aldehydes									
21 *	1-octanal	1008.1	598.85	1.81	39.25 ± 2.89 ^d^	54.46 ± 5.39 ^d^	96.09 ± 1.26 ^c^	162.95 ± 4.42 ^b^	251.27 ± 20.35 ^a^
22 *	2,4-Hexadienal, (E,E)-D	913.2	418.87	1.11	296.28 ± 37.08 ^e^	443.87 ± 4.81 ^d^	662.47 ± 30.12 ^c^	1054.58 ± 13.09 ^b^	1192.19 ± 26.03 ^a^
23 *	2,4-Hexadienal, (E,E)-E	914	420.2	1.46	115.63 ± 10.49 ^d^	138.87 ± 5.59 ^d^	218.53 ± 17.80 ^c^	574.92 ± 14.19 ^b^	687.30 ± 43.51 ^a^
24 *	Heptanal-D	899	396.31	1.34	357.97 ± 3.33 ^e^	651.17 ± 21.58 ^d^	1104.02 ± 38.50 ^c^	1390.65 ± 10.17 ^b^	1784.99 ± 41.06 ^a^
25 *	Heptanal-M	898.1	394.98	1.69	90.77 ± 14.78 ^d^	137.55 ± 6.20 ^d^	336.83 ± 8.83 ^c^	502.34 ± 6.33 ^b^	912.33 ± 68.00 ^a^
26 *	Furfural-D	831.6	309.88	1.32	68.09 ± 1.61 ^e^	128.83 ± 1.52 ^d^	292.52 ± 4.70 ^b^	148.97 ± 4.79 ^c^	640.03 ± 15.64 ^a^
27 *	Furfural-M	830.9	309.11	1.08	13.58 ± 2.06 ^c^	15.17 ± 1.26 ^c^	29.17 ± 2.32 ^b^	14.53 ± 1.09 ^c^	118.27 ± 1.37 ^a^
28 *	3-methyl butanal	642.4	163.55	1.19	856.01 ± 9.59 ^b^	898.84 ± 23.30 ^a^	809.71 ± 5.88 ^c^	683.84 ± 10.48 ^e^	714.02 ± 13.64 ^d^
29 *	Nonanal	1103.8	785.05	1.48	111.16 ± 3.57 ^d^	141.64 ± 12.84 ^c^	177.60 ± 20.71 ^b^	196.66 ± 8.64 ^b^	233.18 ± 17.73 ^a^
Ketones									
30	2-heptanone-D	889.1	381.71	1.26	246.81 ± 8.98 ^e^	711.22 ± 26.46 ^d^	969.06 ± 53.23 ^c^	1140.10 ± 22.52 ^b^	1319.86 ± 66.21 ^a^
31	2-heptanone-M	887.2	379.05	1.62	35.93 ± 5.20 e	118.32 ± 11.44 ^d^	193.73 ± 16.75 ^c^	255.31 ± 4.90 ^b^	350.98 ± 29.94 ^a^
32	2,3-pentanedione	693.8	187.33	1.2	364.53 ± 6.07 ^d^	877.30 ± 50.39 ^c^	1288.15 ± 8.71 ^b^	1331.78 ± 18.72 ^b^	1384.61 ± 20.99 ^a^
33	3-Pentanone	694.9	188.1	1.35	241.26 ± 6.66 ^d^	762.29 ± 22.41 ^c^	851.37 ± 19.10 ^b^	1042.54 ± 17.77 ^a^	1076.50 ± 46.20 ^a^
34	2-Butanone	578.8	139.25	1.24	1366.58 ± 94.32 ^a^	1016.85 ± 34.25 ^b^	1030.21 ± 0.98 ^b^	801.57 ± 30.33 ^c^	1288.02 ± 100.67 ^a^
Sulfur compounds									
35	Ethyl 3-(methylthio)propanoate	1095.2	766.21	1.2	79.49 ± 5.16 ^c^	82.75 ± 2.83 ^c^	99.90 ± 10.23 ^c^	163.82 ± 4.27 ^b^	786.64 ± 73.89 ^a^
36 *	1-Propene, 3,3′-thiobis-	852.6	334.37	1.11	3728.31 ± 139.28 ^b^	4174.29 ± 83.63 ^a^	3367.34 ± 182.63 ^c^	3903.09 ± 51.87 ^b^	2524.93 ± 114.85 ^d^
37	1-Propanethiol	611.9	151.4	1.17	91.29 ± 3.63 ^e^	174.81 ± 7.13 ^d^	195.80 ± 0.45 ^b^	184.88 ± 1.97 ^c^	224.60 ± 0.26 ^a^
38	2-Propanethiol	575.1	137.95	1.14	258.35 ± 4.24 ^d^	290.80 ± 7.92 ^c^	353.29 ± 8.01 ^b^	251.08 ± 7.98 ^d^	427.69 ± 2.85 ^a^
Pyrazines									
39 *	methylpyrazine	819.3	296.35	1.38	44.91 ± 7.23 ^b^	33.93 ± 3.46 ^b^	35.43 ± 3.51 ^b^	41.72 ± 2.25 ^b^	250.89 ± 7.43 ^a^
Heterocyclic class									
40 *	2-pentyl furan	991.7	568.73	1.24	38.42 ± 4.27 ^e^	72.85 ± 2.15 ^d^	109.52 ± 13.66 ^c^	161.39 ± 7.59 ^b^	284.14 ± 35.39 ^a^
Alkanes									
41	Hexane, 2,3,5-trimethyl-	817.9	294.8	1.65	56.67 ± 3.82 ^b^	55.87 ± 6.59 ^b^	57.34 ± 2.70 ^b^	60.22 ± 10.71 ^b^	1171.41 ± 94.94 ^a^

Note: ^a–e^ Means in the same indexes with different letters differ significantly (*p* < 0.05). * are key volatile compounds affecting organoleptic properties.

## Data Availability

The original contributions presented in the study are included in the article/Supplementary Materials, and further inquiries can be directed to the corresponding authors.

## Supplementary Materials

The following supporting information can be downloaded at https://www.mdpi.com/article/10.3390/foods14040556/s1: Table S1: The major nutrition profiles of sausage (g/100 g); Table S2: The sausage colors; Table S3: The sausage DSC.
