# Supplementary material for: Characterization of Fusarium venenatum Mycoprotein-Based Harbin Red Sausages
Source: Foods. 2025 Feb 7;14(4):556. doi: 10.3390/foods14040556 (PMC11854285; doi:10.3390/foods14040556)
Supplement: Supplementary file 1 [file foods-14-00556-s001.zip › foods-3435330-supplementary.pdf]

Table S1: The major nutrition profiles of sausage (g/100 g)

Sample	protein	fat	water	ash
MP1	16.13±0.53^{c}	14.81±0.43^{a}	64.46±0.62^{a}	3.61±0.01^{a}
MP2	16.67±0.60^{c}	14.17±0.36^{a}	62.45±0.74^{b}	3.21±0.19^{ab}
MP3	16.73±0.17^{c}	12.76±0.79^{b}	61.56±0.46^{b}	2.80±0.36^{b}
MP4	21.76±0.39^{b}	9.03±1.01^{b}	57.46±1.05^{c}	3.26±0.18^{ab}
MP5	23.23±0.28^{a}	11.96±0.57^{b}	50.73±0.94^{d}	2.88±0.19^{d}

Note: $^{a–e}$ Means in the same indexes with different letters differ significantly ($p < 0.05$)

Table S2: The sausage of colour

Sample	L*	a*	b*
MP1	43.87±0.41^{d}	6.42±0.33^{a}	5.33±0.55^{d}
MP2	47.00±0.81^{c}	8.33±0.54^{a}	7.10±0.88^{c}
MP3	53.58±0.83^{b}	5.88±0.48^{b}	11.85±0.30^{b}
MP4	52.67±0.61^{b}	6.06±0.45^{b}	12.70±0.89^{b}
MP5	56.07±1.03^{a}	4.23±0.50^{c}	16.88±0.33^{a}

Note: $^{a–e}$ Means in the same indexes with different letters differ significantly ($p < 0.05$)

Table S3: The sausage of DSC

Sample	water content (%)	frozen water content (%)	non-frozen water content (%)	starting point (°C)	peak (°C)
MP1	64.46±0.62[a]	25.98±1.86[a]	38.48±2.16[ab]	0.30±0.14[a]	0.80±0.14[a]
MP2	62.45±0.74[b]	28.93±2.91[a]	34.06±2.68[abc]	0.35±0.70[a]	0.70±0.10[a]
MP3	61.56±0.46[b]	21.37±4.53[a]	40.19±4.36[a]	-5.85±0.49[c]	-3.30±1.13[b]
MP4	57.46±1.05[c]	26.82±4.49[a]	30.64±5.73[bc]	-4.40±0.57[b]	-1.75±1.16[b]
MP5	50.43±0.94[d]	24.59±0.13[a]	26.14±0.30[c]	-5.65±0.64[c]	-2.70±0.28[c]

Note: [a–e] Means in the same indexes with different letters differ significantly ($p < 0.05$)
